# A Single Session of Slackline Training Induces Rapid, Task‐Specific Balance Improvements and Elevated Resting‐State Beta Band Power

**DOI:** 10.1002/ejsc.70205

**Published:** 2026-06-16

**Authors:** Rouven Kenville, Dennis Groß, Maximilian Helbich, Hendrik Behrens, Patrick Ragert, Tom Maudrich

**Affiliations:** ^1^ Department of Movement Neuroscience Faculty of Sport Science University of Leipzig Leipzig Germany; ^2^ Department of Neurology Max Planck Institute for Human Cognitive and Brain Sciences Leipzig Germany

**Keywords:** motor acquisition, postural control, resting‐state EEG, somatosensory‐evoked potentials

## Abstract

Balance training underpins both sports performance and rehabilitation, while its efficacy depends on the amount of training and task difficulty. Here, we characterized neurophysiological changes accompanying the earliest phase of slackline‐specific balance acquisition within a closely matched active‐control design. For this purpose, 35 healthy, slackline‐naïve adults were randomized to a slackline intervention or a time‐matched active control. Before and after training, participants completed slackline single‐leg stance with eyes open (SL‐EO) and eyes closed (SL‐EC), resting‐state electroencephalography (EEG), and tibial‐nerve somatosensory‐evoked potentials (SEP). EEG band‐specific power was quantified after aperiodic correction and resulting change scores were analyzed with non‐parametric mixed models. The intervention group showed larger gains in SL‐EO than control, whereas SL‐EC showed no between‐group difference. Resting‐state EEG exhibited a band‐specific pattern with a greater post‐training increase in beta power in the intervention group, whereas alpha and theta showed no selective group effects. SEP amplitudes did not change pre‐post in either group. Finally, within the intervention group, beta power change did not correlate with individual performance gains. Overall, the present study revealed that resting‐state beta power was sensitive to the earliest phase of slackline‐specific balance acquisition, whereas tibial‐nerve SEP amplitudes remained unchanged. These findings suggest that resting beta power may capture acute post‐practice sensorimotor network changes after a single session of slackline training. Future work should assess generalizability across tasks and populations, combine task‐based EEG with resting measures to link brain state to on‐task control, and track if SEP changes arise over practice to refine models of balance learning.

## Highlights

- Resting‐state EEG showed a selective post‐training increase in beta‐band power after a single session of slackline training, whereas theta and alpha power showed no comparable intervention‐specific effects.
- Short‐latency tibial‐nerve somatosensory‐evoked potential amplitudes remained unchanged, suggesting that acute slackline learning may not immediately alter stimulus‐locked somatosensory processing.
- Changes in resting‐state beta power were not significantly associated with individual balance improvements, indicating that beta power may reflect training‐related sensorimotor network state rather than direct performance gains.

## 1 Introduction

Balance training is a cornerstone of both sports performance enhancement and rehabilitation, and its effectiveness appears to depend on both dose and the degree to which balance is challenged (Sherrington et al. 2017). Across settings, from athletic preparation to clinical practice, there is growing interest in identifying training modalities that place high demands on postural control and thereby induce robust adaptations. Slackline training, which requires maintaining balance over a narrow, compliant webbing, exemplifies such a high‐challenge balance task. Previous work shows that slackline practice elicits pronounced, task‐specific gains in standing time and closely related dynamic tasks (Donath et al. 2017; Giboin et al. 2018). Importantly, even a single session of targeted balance training can temporarily improve balance performance (Muehlbauer et al. 2021). This is consistent with behavioral and neurophysiological accounts of motor skill acquisition, which propose an initial fast learning phase within a single practice session, followed by consolidation and later slow learning with continued practice (Dayan and Cohen 2011; Karni et al. 1998). Even very brief training can induce rapid structural and functional changes. For example, 1 hour of complex balance practice has been shown to produce specific increases in motor cortical gray matter thickness in regions controlling the lower limbs and trunk (Taubert et al. 2016), and micro‐offline performance gains during short rest periods within early practice further support rapid consolidation on the order of seconds (Bönstrup et al. 2020). On this basis, the present study focuses deliberately on the acute timescale, examining changes emerging within a single slackline training session to characterize early behavioral learning and its neural correlates.

Resting‐state brain activity has been used to capture individual differences in neural function related to subsequent motor behavior (Deco et al. 2011; Wu et al. 2014). It is shaped by experience (Lewis et al. 2009), reflects the functional organization of networks that are engaged during task performance, and has been linked to learning and memory outcomes (Lum et al. 2023; Tambini et al. 2010). Spectral resting‐state electroencephalography (EEG) power, in particular, has been proposed as an index of motor aptitude and readiness (Morrone and Pedlar 2024). Recent work in balance paradigms supports this view. Resting‐state alpha power has been shown to predict subsequent learning performance in a complex balance task (Imani and Godde 2025). During balance tasks, increases in frontal, central, and parietal theta power appear to support error monitoring and continuous postural control (Hulsdunker et al. 2015). Challenging postural conditions also modulate beta activity, with higher beta power observed during demanding balance tasks (Ghosn et al. 2020). Together, these findings suggest that theta‐, alpha‐, and beta‐band dynamics provide meaningful neural markers of balance control and its short‐term adaptation. Somatosensory evoked potentials (SEP) offer a second, complementary modality to probe training‐related functional neuroplasticity. SEPs are time‐locked cortical responses to peripheral stimulation and are widely used to assess the integrity, functional organization, and neuroplasticity of the somatosensory system in humans (Macerollo et al. 2018; Passmore et al. 2014). Short‐latency SEP components within the first 100 ms after stimulation are particularly informative, and their amplitudes have been related to motor expertise (Maudrich et al. 2022). Long‐term motor practice, such as athletic training, has been associated with amplitude modulation of specific SEP components in some studies (Bulut et al. 2003; Murakami et al. 2008), whereas others have reported no such differences (Bieru 2015; Yamashiro et al. 2013). Short‐term motor learning can also induce SEP amplitude changes. For example, novel motor tasks have been shown to elicit plastic alterations in early SEP peaks over relatively brief time frames (Andrew, Haavik, et al. 2015; Dancey et al. 2016), although there are also reports of absent short‐term SEP modulation after motor skill learning (Predel et al. 2020).

Building on this background, the present study used a single slackline training session as a controlled model of acute slackline‐specific balance acquisition. Crucially, an active control condition was designed to closely match the slackline intervention in structure, duration, and motor engagement, while minimizing dynamic balance demands by performing the movements on the ground. We expect participants undergoing slackline training to show greater gains in slackline performance than those in the active control group. At the neurophysiological level, we anticipate these behavioral improvements to be accompanied by modulation of resting‐state EEG, with specific training‐related increases in band power (theta, alpha, beta) in the intervention group. Given the mixed evidence on short‐term SEP plasticity, we consider component‐specific changes in short‐latency SEP amplitudes as a further, but more uncertain, marker of rapid balance learning. By linking short‐term behavioral gains in a novel balance task with resting‐state EEG and SEP measures, this study aims to characterize the acute neurophysiological signature of this earliest learning window using two complementary non‐invasive measures within an active‐control design. Combining slackline‐specific performance outcomes with resting‐state EEG, SEP recordings, and EEG‐behavior associations enables the advancement of prior work by isolating specific neurophysiological markers sensitive to acute acquisition during a single session of slackline training.

## 2 Materials and Methods

This study partially re‐analyzes data from a previously published experiment (the previous study is visible in the non‐anonymized version). Specifically, SEP and EEG recordings, as well as the initial balance performance data of 25 participants, were included in the present analyses.

### 2.1 Ethical Statement

The experimental procedures were approved by the Ethics Committee of the Medical Faculty at (the specific Ethics Committee and reference number are visible in the non‐anonymized version). All participants provided written informed consent prior to their inclusion in the study, and all procedures complied with the principles outlined in the Declaration of Helsinki.

### 2.2 Participants

A total of 41 healthy participants (15 female) were enrolled in the present study (age: 23.7 ± 2.6, mean ± SD). None of the participants had previous experience with slacklining. Four participants were excluded from the dataset due to technical issues that rendered their SEP and EEG recordings unusable. Furthermore, two participants were removed from the analyses after being identified as outliers in the behavioral data. Outliers were determined as values exceeding 1.5 times the interquartile range for the respective variable. The final sample comprised 35 participants, who were randomly allocated to one of two groups: (1) intervention group (IG; *n* = 19) and (2) control group (CG; *n* = 16). Demographic and anthropometric data, as well as between‐group comparisons, are presented in Table 1.

**TABLE 1:** Demographic and anthropometric data.

Variable	Intervention (IG)	Control (CG)	Between group comparison
Age (yrs.)	23.5 ± 2.1	23.1 ± 2.7	*p* = 0.483
Height (cm)	175.1 ± 8.9	177.4 ± 11.4	*p* = 0.551
Weight (kg)	71.2 ± 10.3	70.1 ± 12.0	*p* = 0.855
Physical activity (h/week)	2.6 ± 2.4	6.0 ± 4.1	*p* = 0.011*

*Note:* Values are displayed as mean ± SD. * indicates a statistically significant difference between groups.

Individual footedness was determined using one item from the Waterloo Footedness Questionnaire (Elias et al. 1998) that is most relevant for single‐leg balance tasks: *“If you were to balance on one foot, which foot would you use?”.* Based on participant responses, 23 individuals were classified as right‐leg dominant and 12 as left‐leg dominant. For analyses involving lateralized measures, comparisons were conducted between each participant's dominant and non‐dominant leg rather than between right and left sides.

### 2.3 Procedure

**FIGURE 1:**
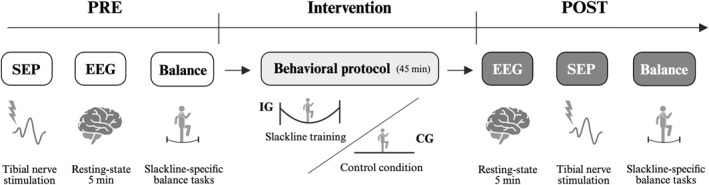
Experimental timeline. All participants completed pre‐ and post‐measurements of somatosensory evoked potentials (SEP), resting‐state EEG, and slackline‐specific balance performance. Between these sessions, participants underwent a behavioral protocol consisting of either slackline training for the intervention group (IG) or a control condition for the control group (CG). The entire experimental session lasted approximately 150 min.

All participants first underwent SEP measurements, followed by a 5‐min resting‐state EEG recording. Subsequently, balance performance was evaluated using slackline‐specific balance tasks. Thereafter, participants completed a behavioral protocol (intervention or control protocol). Following the behavioral protocol, both resting‐state EEG and SEP measurements were repeated, and balance performance was reassessed. A detailed description of all experimental modalities is provided in the following sections. Please refer to Figure 1 for a graphical overview of the experiment.

#### 2.3.1 Somatosensory‐Evoked Potentials (SEP)

Each participant was fitted with a 32‐channel EEG cap (Easycap GmbH, Germany) arranged according to the international 10–20 system, with Cz positioned at the vertex. To enable simultaneous SEP and EEG recordings, the cap was modified by cutting two circular openings (2.5 cm in diameter): one at Fz and another located 2 cm posterior to Cz (referred to as Cz’). Somatosensory‐evoked potentials (SEPs) were recorded using a Nihon Kohden Neuropack X1 system (Nihon Kohden Corp., Japan) during electrical stimulation of the tibial nerve. To reduce electrode impedance, the skin at Fz and Cz’ was gently abraded using OneStep AbrasivePlus Gel prior to electrode placement. Conductive paste was applied to affix the recording electrode at Cz’ and the reference electrode at Fz, with all impedances maintained below 5 kΩ. Participants were positioned supine on a standard massage table. Bilateral tibial nerve stimulation was performed separately using block electrodes placed just below the medial malleolus. SEP signals were sampled at 5120 Hz with an online band‐pass filter of 5–1500 Hz. Stimulation intensity was individually set to 2 mA above the motor threshold, defined as the minimum current required to elicit a visible muscle twitch. For each leg (dominant and non‐dominant), 400 square‐wave pulses (0.2 ms duration) were delivered at a frequency of 3 Hz during the pre‐measurement (PRE). Identical SEP recordings were repeated after the behavioral experiment (POST).

To ensure consistent electrode placement across sessions, stimulation sites were marked with a permanent marker, and stimulation intensity was kept constant between PRE and POST measurements. Averaged SEP waveforms were analyzed to identify the latencies and amplitudes of the N30, P40, N50, and P60 components. Peak‐to‐peak amplitudes between N30‐P40, P40‐N50, and N50‐P60 were extracted for both PRE and POST conditions. Relative changes in SEP amplitudes were then calculated as percentage differences between POST and PRE values for each component and stimulation side (dominant and non‐dominant leg). The resulting change scores were entered into the statistical analyses.

#### 2.3.2 EEG Recordings

During resting‐state EEG acquisition, participants sat comfortably in front of a standard computer monitor and were instructed to fixate on a white cross presented on a black background for 5 minutes. EEG signals were recorded using a mobile 32‐channel amplifier (LiveAmp, Brain Products GmbH, Germany) with active electrodes mounted on the custom 32‐channel EEG cap described above. Conductive gel (SuperVisc High‐Viscosity Electrolyte Gel) was applied to each electrode site to maintain low impedance, which was kept below 10 kΩ throughout data collection. The EEG signal was transmitted wirelessly to a workstation via the Bluetooth module integrated into the LiveAmp system. Data were sampled at 500 Hz with an input impedance greater than 100 MΩ and a common mode rejection ratio (CMRR) exceeding 80 dB. A 1–100 Hz online band‐pass filter was applied during acquisition.

#### 2.3.3 EEG Preprocessing and Spectral Analysis

EEG data were preprocessed in MATLAB (R2025b, The MathWorks Inc., Natick, USA) using the *EEGLAB* toolbox (Delorme and Makeig 2004) in combination with custom‐written routines. The continuous signals were first filtered using a 1–100 Hz band‐pass filter and a 48–52 Hz notch filter to attenuate power line interference. Channel‐ and epoch‐level noise was detected via the *clean_rawdata* plugin in *EEGLAB* and subsequently verified through visual inspection by an experienced researcher before removal. After artifact rejection, all channels were re‐referenced to the common average. Independent component analysis (ICA) was then applied using the *runica* algorithm to further isolate residual artifacts. Components associated with eye blinks, muscle activity, or other non‐neural sources were identified with the *ICLabel* plugin (Pion‐Tonachini et al. 2019). Components labeled as artifacts by both the algorithm and visual inspection were excluded from the data.

We estimated power spectral density (PSD) from cleaned resting‐state EEG using Welch's method (Hann window: 512 samples, 50% overlap, nfft = 1024). PSDs were treated in absolute units (μV^2^ or μV^2^/Hz) throughout. To isolate oscillatory power from the 1/f‐like background, we employed FOOOF v1.1.1 ^31^ within a combined Matlab and Python (v3.11.14) workflow to parametrize and subtract the aperiodic component. The model used fixed aperiodic mode (no spectral knee), peak width limits between 1 and 8 Hz, a maximum number of 3 peaks, and a minimum peak height of 0.1. For each participant, channel, and time‐point, we obtained (1) the aperiodic fit and (2) the flattened spectrum. We then constructed a positive‐excess spectrum, that is, power strictly above the aperiodic baseline, by converting the flattened (log) output back to linear units and setting negative deviations to zero (Donoghue et al. 2020; Cross et al. 2022). Band‐limited oscillatory power (theta 4–7 Hz; alpha 8–12 Hz; beta 13–30 Hz) was then computed as the area under the excess spectrum within each band, yielding absolute, non‐negative and aperiodic‐adjusted band powers. For regional summaries, we averaged channel‐wise band powers within two a priori ROIs: fronto‐central (Fp1, Fp2, F3, F4, FC1, FC2) and centro‐parietal (Cz, CP1, CP2, C3, C4), both based on previous related research (Hulsdunker et al. 2015; Slobounov et al. 2008). Finally, within‐subject absolute change scores (POST–PRE) per ROI and band were used for further statistical analyses.

#### 2.3.4 Slackline‐Specific Balance Performance

After completing SEP and EEG recordings, participants performed two slackline‐specific balance tasks: single‐leg stance with eyes open (SL‐EO) and single‐leg stance with eyes closed (SL‐EC) (Giboin et al. 2019; Magon et al. 2016). Each task was executed on both the dominant (SL‐EO_D_ and SL‐EC_D_) and non‐dominant leg (SL‐EO_ND_ and SL‐EC_ND_). Two slackline setups were employed. The first consisted of a 4‐m slackline (width: 5 cm) mounted on a rigid metal frame (Slackstar, Braun GmbH, Germany) at a height of 55 cm, with small platforms on either end to allow for safe mounting and dismounting. This setup was used for the SL‐EO condition. The second setup utilized a GIBOARD (Gibbon, ID Sports GmbH, Germany), a 1‐m slackline mounted at a height of 14 cm, which was used for the SL‐EC condition to minimize injury risk during eyes‐closed performance. In each condition, participants completed two trials, and the better of the two scores was used for analysis. This procedure helped reduce the impact of accidental or unrepresentative failures (e.g., immediate loss of balance) while limiting task exposure, thereby preserving the assessment of genuine initial performance in this task‐naïve cohort (Magon et al. 2016). For both SL‐EO and SL‐EC, participants were instructed to maintain a single‐leg stance in the middle of the slackline for as long as possible without touching the ground. The score was defined as the maximum duration of stable balance. All tasks were performed consecutively with 15‐s rest intervals between trials. Two experimenters stood nearby throughout all tasks to prevent potential injury in the event of a fall. The order of tasks and starting leg were randomized to counterbalance potential sequence effects. Balance performance was assessed before (PRE) and after (POST) the behavioral intervention. Percentage change scores between POST and PRE were calculated and entered into the statistical analyses.

#### 2.3.5 Behavioral Protocol

Participants in the intervention group (IG) completed a single session of supervised slackline‐specific balance training. The session consisted of a standardized lower‐body warm‐up followed by a sequence of single‐leg balance exercises performed on a slackline. To ensure comparability with previous research, the standardized training protocol (“TE 2”) described by Giboin et al. (2019) was adopted. All exercises were performed on the same 4‐m slackline (width: 5 cm) used during balance performance assessments, mounted on a rigid metal frame at a height of 55 cm. The total duration of the balance training session was approximately 45 min. In contrast, the active control group (CG) performed the identical sequence of balance exercises with the slackline (length: 4 m; width: 5 cm) placed directly on the ground to minimize dynamic balance demands while maintaining comparable task structure and training duration. Exercise order and total session duration were identical between groups.

#### 2.3.6 Statistical Analysis

Statistical analyses were conducted using RStudio (version 2025.9.1.401). Normality of all relevant variables was first assessed using Shapiro‐Wilk tests (alpha = 0.05). As most behavioral outcome measures deviated from normality, non‐parametric methods were applied. Group comparisons of anthropometric and demographic data were performed using pairwise Mann–Whitney‐U tests. To examine intervention‐induced changes in outcome parameters, three separate non‐parametric mixed ANOVAs were computed using the *ARTool* package, which implements the Aligned Rank Transform for factorial models (Wobbrock et al. 2011; Kay et al. 2025). All ANOVAs were modeled on change scores (POST‐PRE) of the respective dependent variables. Change scores were analyzed to account for baseline differences between groups and to isolate intervention‐related changes independent of initial levels. For slackline‐specific balance performance, the model included the between‐subject factor GROUP (IG, CG) and the within‐subject factors SIDE (D, ND) and TEST (SL‐EO, SL‐EC). For SEP data, the model included GROUP (IG, CG) as the between‐subject factor and SIDE (D, ND) and COMPONENT (N30‐P40, P40‐N50, N50‐P60) as within‐subject factors. For EEG measures, the model included GROUP (IG, CG) as the between‐subject factor and BAND (theta, alpha, beta) and ROI (fronto‐central, centro‐parietal) as within‐subject factors. Pairwise post‐hoc comparisons for each model were conducted using the ART‐C contrast testing procedure, applying false discovery rate (FDR) correction to control for multiple comparisons (Elkin et al. 2021). Finally, within IG, Spearman rank correlations were conducted to assess associations between change scores in SL‐EO performance and beta power. Correlations were computed separately for each combination of SIDE (dominant, non‐dominant) and ROI (fronto‐central, centro‐parietal).

## 3 Results

### 3.1 Sample Characteristics

No significant group differences were observed between the intervention group (IG) and control group (CG) regarding age (*U* = 173.5, *p* = 0.483, *r*_bs_ = 0.14), height (*U* = 133.5, *p* = 0.551, *r*_bs_ = −0.12), or body weight (*U* = 158.0, *p* = 0.855, *r*_bs_ = 0.04). However, a significant difference emerged for weekly physical activity levels (*U* = 75.0, *p* = 0.011, *r*_bs_ = −0.51), with CG reporting higher physical activity compared to IG (6.0 ± 4.1 vs. 2.6 ± 2.4 h/week).

### 3.2 Slackline‐Specific Balance Performance

**FIGURE 2:**
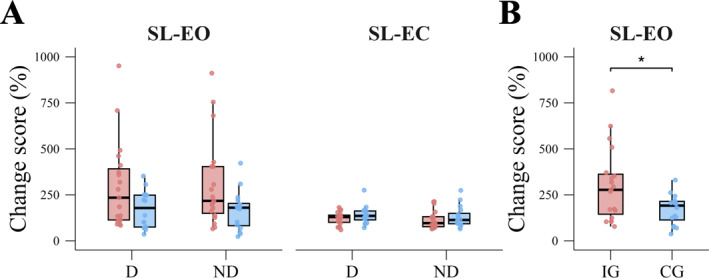
Slackline‐specific balance assessment. (A) Group comparison of change scores in the slackline‐specific balance assessment for the single‐leg stance with eyes open (SL‐EO) and single‐leg stance with eyes closed (SL‐EC) conditions, performed with the dominant (D) and non‐dominant (ND) leg. Red boxes represent the intervention group (IG), blue boxes the control group (CG). (B) Significant GROUP × TEST interaction for SL‐EO collapsed across SIDE (D, ND). Asterisk denotes a significant between‐group difference (*p* < 0.05).

A significant GROUP × TEST interaction was observed (*F*_(1, 99)_ = 16.33, *p* = 0.001, *η*_p_^2^ = 0.142). Post‐hoc comparisons indicated that IG exhibited greater performance improvements than CG during the SL‐EO condition (302.8 ± 233.7% vs. 169.4 ± 101.3%, *t*_(70.9)_ = 2.36, *p* = 0.042). In addition, significant main effects were found for GROUP (*F*_(1, 33)_ = 4.40, *p* = 0.044, *η*_p_^2^ = 0.118) and TEST (*F*_(1, 99)_ = 28.75, *p* < 0.001, *η*_p_^2^ = 0.225). Across conditions, IG showed overall higher change scores than CG (210.7 ± 190.9% vs. 152.5 ± 81.7%, *t*_(33)_ = 2.10, *p* = 0.044), and participants improved more in SL‐EO compared to SL‐EC (241.8 ± 195.9% vs. 126.3 ± 47.5%, *t*_(99)_ = 5.36, *p* < 0.001). A comprehensive overview of the behavioral findings is provided in Figure 2.

### 3.3 EEG Power

**FIGURE 3:**
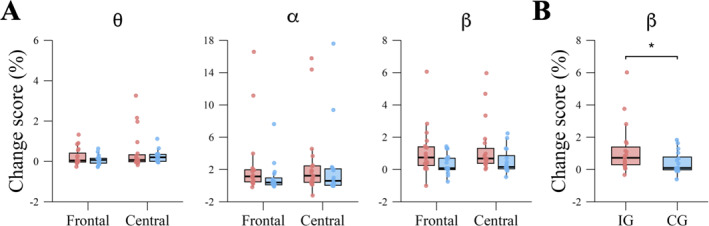
EEG spectral power. (A) Group comparison of change scores in EEG spectral power for the theta (*θ*), alpha (*α*), and beta (*β*) frequency bands across frontal and central regions of interest (ROIs). Red boxes represent the intervention group (IG), blue boxes the control group (CG). (B) Significant GROUP × BAND interaction for beta power averaged across ROIs. Asterisk denotes a significant between‐group differences (*p* < 0.05).

A significant GROUP × BAND interaction was revealed (*F*_(2, 165)_ = 6.01, *p* = 0.003, *η*_p_^2^ = 0.068). Post‐hoc comparison indicated that beta power increased more in IG compared to CG (1.2 ± 1.5% vs. 0.4 ± 0.7%, *t*_(58.1)_ = 2.66, *p* = 0.017). Furthermore, significant main effects for GROUP (*F*_(1, 33)_ = 4.57, *p* = 0.04, *η*_p_^2^ = 0.122), BAND (*F*_(2, 165)_ = 31.29, *p* < 0.001, *η*_p_^2^ = 0.275) and ROI (*F*_(1, 165)_ = 9.53, *p* = 0.002, *η*_p_^2^ = 0.055) were found. Across frequency bands, IG showed higher change scores than CG (1.4 ± 2.8% vs. 0.8 ± 2.2%, *t*_(33)_ = 2.14, *p* = 0.040). Change scores in alpha power were higher compared to theta power (2.2 ± 4% vs. 0.3 ± 0.6%, *t*_(165)_ = 7.79, *p* < 0.001) and compared to beta power (2.2 ± 4% vs. 0.8 ± 1.3%, *t*_(165)_ = 2.67, *p* = 0.008). Furthermore, change scores in beta power were higher compared to theta power (0.8 ± 1.3% vs. 0.3 ± 0.6%, *t*_(165)_ = 5.11, *p* < 0.001). Finally, the centro‐parietal ROI showed higher change scores than the fronto‐central ROI (1.3 ± 2.9% vs. 0.9 ± 2.2%, *t*_(165)_ = 3.09, *p* = 0.002). A comprehensive overview of EEG power results is provided in Figure 3.

### 3.4 Somatosensory‐Evoked Potentials (SEP)

For SEP amplitude changes, no significant main effects or interactions were observed (all *p* > 0.05).

### 3.5 Spearman Correlations

**FIGURE 4:**
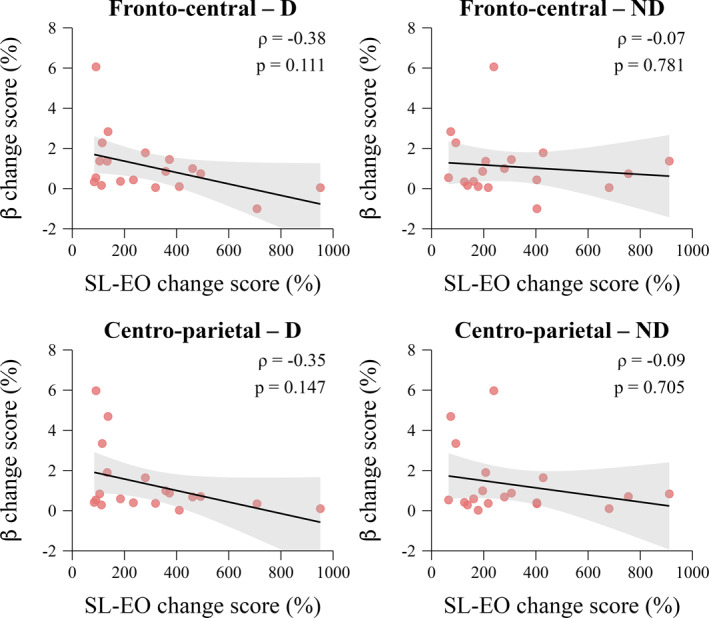
Associations between EEG beta power changes and balance performance. Spearman correlations between changes in EEG beta power and slackline‐specific balance performance (SL‐EO) for the intervention group (IG). Each panel represents a separate correlation for the frontal (fronto‐central) and central (centro‐parietal) regions of interest (ROIs), and for the dominant (D) and non‐dominant (ND) leg. Shaded areas indicate 95% confidence intervals of the trendline.

Within the intervention group (IG), no significant correlations were found between changes in beta power within the fronto‐central ROI and improvements in SL‐EO performance for either the dominant (*n* = 19, *r*_s_ = −0.377, 95% CI [−0.761, 0.130], *p* = 0.111) or non‐dominant leg (*n* = 19, *r*_s_ = −0.068, 95% CI [−0.541, 0.448], *p* = 0.781). Similarly, no significant associations were observed between beta power changes in the centro‐parietal ROI and performance improvements in SL‐EO for either the dominant (*n* = 19, *r*_s_ = −0.346, 95% CI [−0.736, 0.125], *p* = 0.147) or non‐dominant leg (*n* = 19, *r*_s_ = −0.093, 95% CI [−0.556, 0.360], *p* = 0.705). The analyzed correlations are summarized in Figure 4.

## 4 Discussion

With the present study, we aimed to investigate neurophysiological markers accompanying the earliest phase of slackline‐specific balance acquisition. Here, we observed participants in the intervention group to show markedly greater improvements in SL‐EO than those in the active control group, whereas no between‐group or pre‐post differences were observed for SL‐EC. At the neurophysiological level, pre‐post changes in resting‐state EEG revealed a more pronounced increase in beta power in the intervention group relative to the control group. To probe potential associations between behavioral and EEG measures, we correlated individual resting‐state beta power changes with performance gains but did not observe significant relationships. Short‐latency SEP amplitudes did not show significant pre‐post changes in either group. All findings and their implications are discussed in detail below.

The behavioral findings confirm that the present paradigm successfully induced the expected task‐specific improvement in slackline performance. This is in line with previous work showing that short bouts of balance training can yield rapid improvements. Muehlbauer et al. (2021) reported that a single unilateral balance training session improved ipsilateral and contralateral balance performance in healthy young adults, independent of the specific practice condition. In older adults, Alizadehsaravi, et al. (2020) observed that unipedal standing performance improved after a single session of balance training, with only modest additional benefits across further sessions. More specifically, the behavioral results validate the experimental contrast, with the slackline‐trained group improving more than the active control group and gains emerging only for SL‐EO, which mirrors the absence of eyes‐closed practice in both groups. This is consistent with a meta‐analytical review concluding that slackline training predominantly leads to meaningful, task‐specific improvements in outcomes closely related to the training content, while transfer to standard static and dynamic balance tests is limited (Donath et al. 2017). Similarly, it has been shown that 3 months of slackline training produce large slackline‐specific performance gains but fail to improve one‐legged stance with eyes open or closed on a force plate (Giboin et al. 2018). Reversing the direction of transfer, Volery et al. (2017) reported specific effects on a balance disc test, with improvements confined to participants who completed conventional sensorimotor training, whereas those who trained on a slackline did not show any performance gains. The lack of intervention effects on SL‐EC is also consistent with a context‐specific view of balance learning. In our protocol, all balance training exercises were performed with eyes open. Consequently, neural and behavioral adaptations induced by balance training were acquired in a visual context that was absent in the SL‐EC balance task. Removing visual input is known to change the relative weighting of visual, vestibular, and somatosensory information and to alter postural control strategies (Yelnik et al. 2015). It is therefore plausible that the improvements observed for SL‐EO do not generalize to a condition that relies more heavily on non‐visual cues. Even in longer‐term slackline interventions, transfer to closed‐eye conditions has been inconsistent, with some studies reporting improvements under eyes‐closed conditions of clinical tests after intensive slackline practice (Dordevic et al. 2017) and others finding no such effects (Giboin et al. 2018). Taken together, these findings align with the long‐standing view that balance is not a single, general capacity, but rather a constellation of task‐specific skills (Giboin et al. 2015).

At the neurophysiological level, the most prominent effect was a stronger increase in resting‐state beta power in the intervention group compared to the control group, alongside general band‐ and region‐specific change patterns. Across groups, alpha‐band change scores were larger than those in beta and theta, and changes tended to be more pronounced over centro‐parietal than fronto‐central sites. The group difference, however, was most evident in the beta band, which aligns with previous work linking beta oscillations to motor practice and learning. Prior work indicates that practicing a visuomotor task can enhance beta power at rest and modify beta modulation during movement in healthy adults (Moisello et al. 2015). Related work has shown that beta modulation depth over contralateral sensorimotor areas increases across practice blocks, even when its correlation with behavioral improvements is weak (Nelson et al. 2017). Furthermore, Özdenizci et al. (2016) showed that the configuration of resting‐state beta power predicts individual adaptation rates in a force‐field reaching task. Beyond local power, resting‐state beta coherence and connectivity have been associated with motor learning capacity. For example, Wu et al. (2014) demonstrated that beta‐band connectivity at rest predicts individual motor skill acquisition, while further research highlighted that resting‐state beta connectivity between motor and other large‐scale networks is related to subsequent motor learning (Sugata et al. 2020; Titone et al. 2022). From a mechanistic perspective, early slackline learning likely involves rapid recalibration of postural control strategies, including changes in motor coordination and sensory weighting (van Dieen et al. 2015). Slackline training imposes continuous dynamic balance errors that require online integration of visual, vestibular, proprioceptive, and motor information. In this context, an acute post‐training increase in resting‐state beta power may reflect a transient change in sensorimotor network state after intensive error‐driven practice. This interpretation is consistent with accounts proposing that beta oscillations support maintenance or stabilization of the current sensorimotor state (Engel and Fries 2010), and with evidence linking beta activity to reactive balance demands (Ghosn et al. 2020). Still, the origin of motor learning‐related beta increases remains debated but is often linked to changes in cortical excitability and inhibition. Theta‐burst stimulation studies have reported associations between increased beta power and decreased corticospinal excitability (McAllister et al. 2013; Noh et al. 2012). Combined magnetoencephalography (MEG) and pharmacological GABA modulation has shown that enhancing GABAergic tone elevates baseline sensorimotor beta power and alters movement‐related beta dynamics, consistent with the view that increased beta synchrony reflects reduced cortical excitability (Muthukumaraswamy et al. 2013). In the context of motor learning, previous work has suggested that transient post‐training beta increases may reflect adjustments in inhibitory tone that constrain plasticity following intensive practice (Cantarero et al. 2013; Espenhahn et al. 2020). Interestingly, this dissociation is consistent with previous reports. Practice‐related improvements in visuomotor performance have been reported to occur without corresponding correlations with changes in beta power at rest or during movement, and increased beta modulation depth across practice blocks has similarly been observed in the absence of strong EEG‐behavior associations (Moisello et al. 2015; Nelson et al. 2017). These findings were taken to suggest that beta‐band oscillatory dynamics reflect underlying plasticity mechanisms or network state changes that are not directly or linearly expressed in behavioral performance. Our data corroborate this view. Resting‐state EEG changes appear to capture aspects of cortical reorganization following slackline training but do not necessarily scale with the magnitude of behavioral improvement across individuals. Balance performance itself is shaped by multiple interacting factors, including peripheral neuromuscular control, biomechanical strategies, attentional allocation, and fatigue (Horak 2006; Paillard 2012). Recent systematic reviews of mobile brain imaging during balance emphasize that cortical responses are highly context‐dependent and that brain‐behavior relationships are often non‐linear and variable across individuals (Huang and Ferris 2023; Purohit and Bhatt 2022). In this light, the absence of robust correlations between increases in beta activity and balance performance gains in our sample is not unexpected and reinforces the interpretation of beta power as a complementary neural correlate of balance training exposure rather than a direct proxy for balance performance.

In contrast to our EEG findings, short‐latency SEP amplitudes did not show significant changes following balance training in either group. The broader literature illustrates that SEP sensitivity to motor learning is variable and appears to depend on task, dose, and measurement parameters (Maudrich et al. 2022). Short‐term motor learning can modulate early SEP components. For instance, significant changes in early SEP peaks were observed following a novel thumb motor task, and further work from the same group showed that different skilled tasks produce distinct patterns of SEP amplitude modulation (Andrew et al. 2015; Andrew et al. 2015b). On the other hand, Predel et al. (2020) found robust improvements in co‐contraction timing in both young and older adults following a relatively intensive motor training protocol, but no significant changes in cortical SEP amplitudes, despite evidence that baseline SEP characteristics predicted learning success. Studies on long‐term training and athletic expertise likewise report contradictory SEP findings. Several investigations have observed amplitude or latency differences between athletes and non‐athletes or between athletes in different sports (Bulut et al. 2003; Murakami et al. 2008), whereas others found no differences despite comparable methodologies (Bieru 2015; Yamashiro et al. 2021). A recent systematic review of our group concluded that a majority of studies reported SEP differences related to long‐term sport training, while emphasizing notable heterogeneity in stimulation protocols, tasks, and sample characteristics (Maudrich et al. 2022). Taken together, the absence of SEP amplitude changes may indicate that the present acute learning window did not significantly alter early somatosensory afferent processing at the level captured by tibial‐nerve short‐latency SEPs. Since SEPs provide a non‐invasive measure of somatosensory pathway activation and plasticity (Macerollo et al. 2018), unchanged amplitudes suggest that the earliest adaptations to slackline training may have been expressed more strongly at the level of distributed sensorimotor network state than as detectable changes in stimulus‐locked cortical responses.

### 4.1 Limitations

Based on our sample of young, healthy, slackline‐naïve adults, the observed behavioral and neurophysiological effects may not generalize to older individuals, clinical populations, or highly trained athletes. Similarly, the intervention comprised a single slackline training session without any follow‐up assessments. Consequently, our study addresses only short‐term motor learning and does not allow for conclusions to be drawn about retention, consolidation, or longer‐term adaptation. Another consideration concerns the difference in self‐reported weekly physical activity between groups, with higher activity levels in the control group. Crucially, all participants were slackline‐naïve, and our analyses focused on within‐subject change scores and a closely matched active control condition. The observed group differences in slackline performance improvements and beta power changes are therefore unlikely to be explained solely by general fitness differences. Nonetheless, future work could more closely match or explicitly control for habitual activity to further minimize potential confounding. Furthermore, we quantified oscillations as absolute, aperiodic‐adjusted power rather than relative power. Methodological work shows that common band ratios are heavily influenced by broadband/aperiodic structure and thus may not index genuine oscillatory peaks (Donoghue et al. 2020). In addition, relative power mixes oscillatory peaks with the aperiodic 1/f background. Thus, shifts in the 1/f slope, which vary with age and arousal/state, can appear to be band‐specific effects when expressed as proportions (Gao et al. 2017; He 2014; Voytek et al. 2015). By parameterizing and removing the aperiodic component and integrating excess power within specific bands, our pre‐post comparisons target the oscillatory contribution more specifically.

## 5 Conclusion

In conclusion, by embedding acute slackline‐specific balance acquisition within a closely matched active‐control design, the present study helps distinguish neurophysiological changes specifically associated with early slackline learning from more general effects of task exposure, repeated testing, and motor engagement. Future work should pair task‐EEG with resting‐state EEG and simple excitability measures to link brain state to on‐task balance control, while longitudinal assessments of balance performance could pinpoint when SEPs begin to change across training sessions. Collectively, such evidence may refine mechanistic models of early balance learning and clarify how different neurophysiological markers capture distinct aspects of sensorimotor adaptation.

## Author Contributions

R.K, P.R. and T.M. designed the study. R.K., D.G., M.H. and H.B. acquired the data. R.K. and T.M. analyzed the data, and wrote the manuscript. All authors interpreted the data, contributed to the manuscript, reviewed it, approved the content of the final version, and agree to be accountable for all aspects of the work. All persons designated as authors qualify for authorship, and all those who qualify for authorship are listed.

## Funding

The authors have nothing to report.

## Conflicts of Interest

The authors declare no conflicts of interest.

## Data Availability

The data that support the findings of this study are openly available in Figshare at https://figshare.com/articles/dataset/Slacklearn_II/30618410?file=59555771, reference number 10.6084/m9.figshare.30618410.
